# Outlining the Phytoconstituents of Greek Clover Herb Extract and Assessment of Its Effect against Foodborne Infections Caused by *Salmonella typhimurium*

**DOI:** 10.3390/ph17020259

**Published:** 2024-02-18

**Authors:** Jawaher Alqahtani, Walaa A. Negm, Engy Elekhnawy, Moneerah J. Alqahtani, Ehssan Moglad, Sarah Ibrahim, Suzy A. El-Sherbeni

**Affiliations:** 1 Department of Pharmacognosy, College of Pharmacy, King Saud University, Riyadh 11495, Saudi Arabia; mjalqahtani@ksu.edu.sa; 2 Department of Pharmacognosy, Faculty of Pharmacy, Tanta University, Tanta 31527, Egypt; walaa.negm@pharm.tanta.edu.eg (W.A.N.); suzy.elsherbini@pharm.tanta.edu.eg (S.A.E.-S.); 3 Department of Pharmaceutical Microbiology, Faculty of Pharmacy, Tanta University, Tanta 31527, Egypt; 4 Department of Pharmaceutics, College of Pharmacy, Prince Sattam bin Abdulaziz University, Alkharj 11942, Saudi Arabia; e.moglad@psau.edu.sa; 5 Human Anatomy and Embryology Department, Faculty of Medicine, Tanta University, Tanta 31527, Egypt; sara.ibrahim@med.tanta.edu.eg

**Keywords:** antibacterial, chemical profile, GIT infection, inflammation, oxidative stress

## Abstract

Owing to the spread of resistance between pathogenic bacteria, searching for novel compounds with antibacterial activity is essential. Here, we investigated the potential antibacterial activity of Greek clover or *Trigonella foenum-graecum* herb extract on *Salmonella typhimurium* clinical isolates. The chemical profile of the herb was initially determined using LC-ESI-MS/MS, which explored 36 different compounds. Interestingly, the fenugreek extract possessed antibacterial action in vitro with minimum inhibitory concentrations of 64 to 512 µg/mL. The potential mechanism of action was studied by elucidating the effect of the fenugreek extract on the membrane properties of *S. typhimurium* bacteria, including the inner and outer membrane permeability and membrane integrity. Remarkably, the fenugreek extract had detrimental effects on the membrane properties in 40–60% of the isolates. Moreover, the in vivo antibacterial action was studied using a gastrointestinal infection model with *S. typhimurium* bacteria. Interestingly, the fenugreek extract (200 mg/kg) improved the infection outcomes in the tested mice. This was represented by the noteworthy decrease (*p* < 0.05) in the bacterial count in the small intestine and caecum tissues. The survival rate of the fenugreek-extract-treated mice significantly increased compared to the *S. typhimurium*-infected group. Additionally, there was an improvement in the histological and immunohistochemical features of tumor necrosis factor-alpha. In addition, using an ELISA and qRT-PCR, there was an improvement in the proinflammatory and oxidative stress markers in the fenugreek-extract-treated group. Consequently, fenugreek extract should be investigated further on other food pathogens.

## 1. Introduction

Many foodborne infections are triggered by ingesting food and beverages contaminated with pathogenic microorganisms like bacteria, fungi, and parasites [1]. Such foodborne diseases are considered a significant problem to public health worldwide. Salmonellosis infection is regarded as one of the central sources of foodborne diseases, and this infection is brought on by Salmonella species [2].

*Salmonella enterica* serovar *Typhimurium* belongs to the *Enterobacteriaceae* family. Such Gram-negative, facultative anaerobic flagellated rods cannot produce spores [3]. The most common clinical symptom of salmonellosis in animals and humans is gastrointestinal (GIT) disease. However, it may progress to several other disorders like acute septicemia [4].

Antibiotics are frequently used in food animal production in developing countries to promote the well-being and growth of animals [5]. Unfortunately, such countries have major concerns regarding the escalation of antibiotic resistance. Antibiotic resistance can be defined as a failure of antibiotics to affect bacterial progression [6]. Such resistance becomes more complicated if the bacteria are multidrug resistance (MDR). The speedy development of MDR amongst bacteria is triggered by the continued selective pressure and progression of novel survival mechanisms in bacteria [7]. Consequently, there are unlimited struggles to tackle such antibacterial resistance and generate efficient, safe, and eco-friendly antibacterial therapeutic methods [8].

Natural sources, like plants, are considered a treasure as they contain various phytochemical compounds with various potent pharmacological actions [9]. Such plants could be used as therapeutic alternatives to combat infections caused by MDR bacterial pathogens [10]. The phytochemicals of plants could exert their action against bacteria by inhibiting efflux pumps, affecting membrane properties, impairing bacterial virulence, and influencing antibiotic-degrading enzymes [11].

*Trigonella foenum-graecum*, family Fabaceae [12], was traditionally used in ancient times [13]. The seeds, leaves, and sprouts were used as nutraceuticals and in medicinal applications. They are crucial in preventing gastrointestinal disorders and managing diabetes, microbial infections, hypercholesterolemia, and other ailments. Fenugreek has anticancer [14], antioxidant, anti-inflammatory, antimicrobial [15], antilipidemic, demulcent, wound healing [16] and immunological activities [17]. It is a valuable nutraceutical and medicinal plant in various food products [18]. The leaves contain graecunins, which are saponins (glycosides of diosgenin) [19], phenolic acids and flavonoids [20]. Fenugreek seeds are a good source of galactomannan (complex heteropolysaccharides) and saponins such as diosgenin, yamogenin, tigogenin, neotigogens, and gitogenin [19]. The seeds also contain alkaloids (choline and trigonilline), mucilage, flavonoids, phenolic acid derivatives [21], steroids, amino acids, and volatile oils [17]. The fenugreek seed extract possesses antimicrobial and anti-inflammatory activities, as reported previously [22].

This investigation aims to reveal the chemical profile of *Trigonella foenum-graecum* herb extract and discover the potential antibacterial action of fenugreek extract on *S. typhimurium* bacteria in vitro and in vivo.

## 2. Results

### 2.1. Phytochemical Analysis of Trigonella foenum-graecum Herb Using LC-ESI-MS/MS

**Figure 1:**
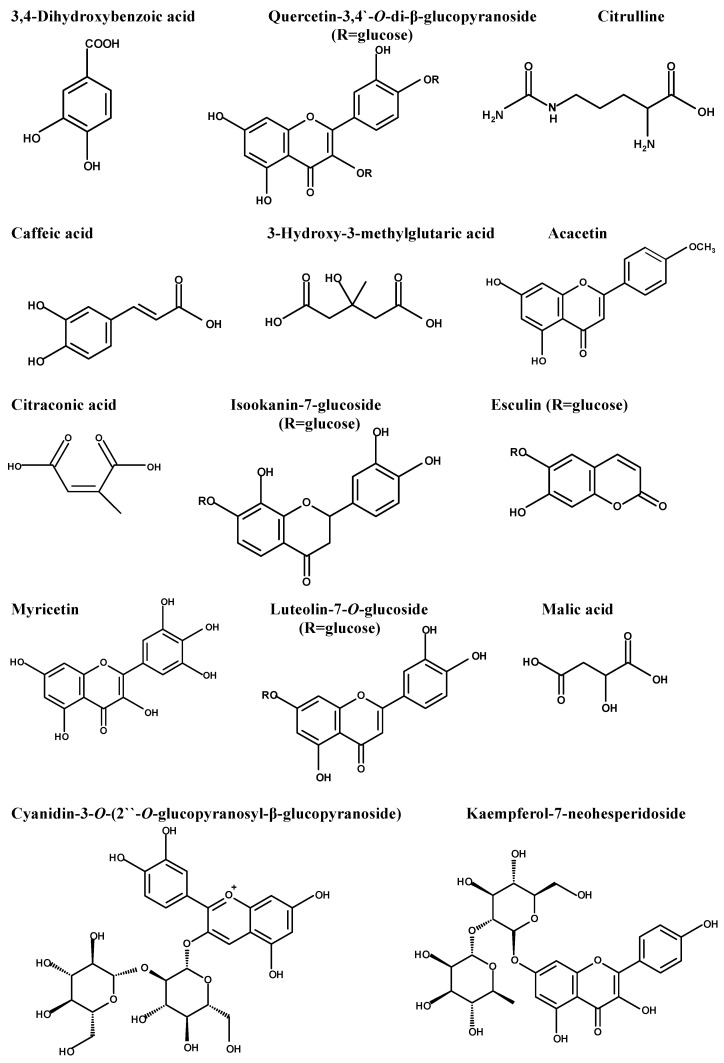
Major compounds detected in *Trigonella foenum-graecum* using LC-ESI-MS/MS.

The diversity of the phytochemical content of fenugreek herb was initially determined using LC-ESI-MS/MS, which recorded the presence of 36 compounds. The major compounds, which were displayed using the negative mode ESI, were 3,4-dihydroxy benzoic acid, quercetin-3,4’-*O*-di-β-glucopyranoside, caffeic acid, citrulline, 3-hydroxy-3-methyl glutaric acid, acacetin, citraconic acid, isookanin-7-glucoside, esculin, myricetin, luteolin-7-*O*-glucoside, and malic acid. Table 1 represents the initial compounds detected using the in-house database and supported by the reported data. Figure 1 represents the major compounds identified in the herb extract using LC-ESI-MS/MS. Figure S1 illustrates the total ion chromatogram in the Supplementary Materials.

### 2.2. Antibacterial Action (In Vitro)

The fenugreek extract showed antibacterial action towards *S. typhimurium* by revealing inhibition zones around the wells in the Muller–Hinton agar (MHA) plates. The broth microdilution technique recorded the minimum inhibitory concentrations (MICs). They ranged from 64 to 512 µg/mL (Table 2).

#### Membrane Properties

**Figure 2:**
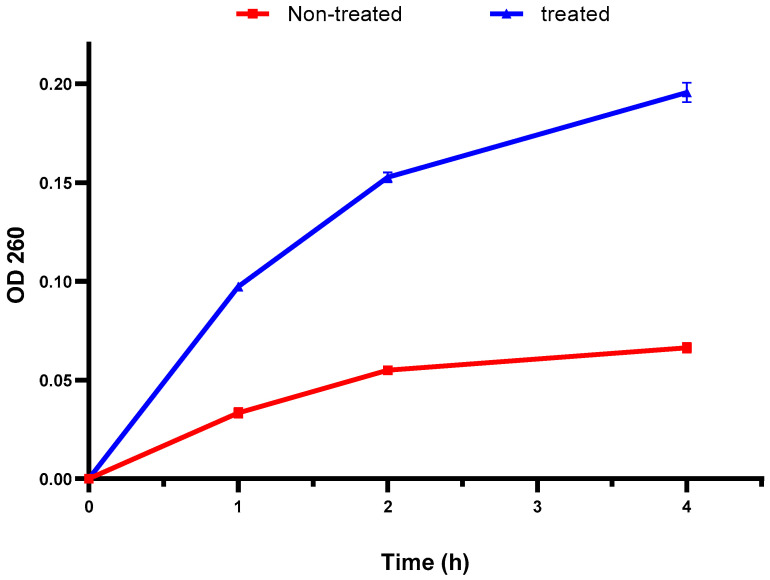
An illustration of the increased discharge of DNA and RNA (with absorbance at 260 nm) after treatment with the fenugreek extract. The red line represents the absorbance before treatment, and the blue line represents the absorbance after treatment with the fenugreek extract. The measured optical density (OD) 260 reveals the release of DNA and RNA from the cellular interior.

The bacterial membrane integrity was explored by elucidating the DNA and RNA discharge from the cellular interior. There was a remarkable increase in the absorbance at 260 nm after treatment with the fenugreek extract in 60% of the isolates (Figure 2). This finding designates the detrimental impact of the fenugreek extract on the bacterial membrane.

**Figure 3:**
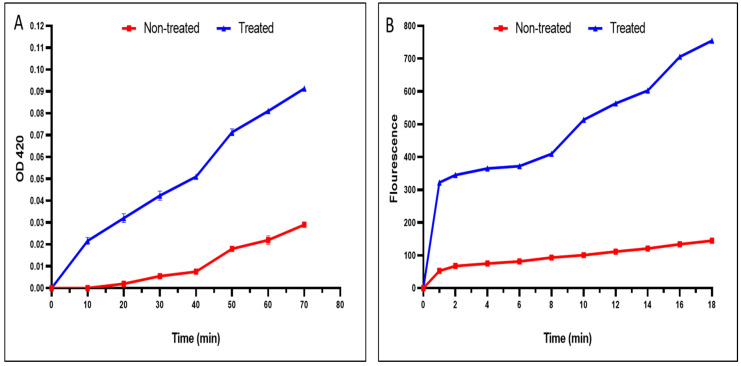
An illustration of the damage caused by the fenugreek extract on the (**A**) inner and (**B**) outer membrane permeability. The red line represents the absorbance before treatment, and the blue line represents the absorbance after treatment with the fenugreek extract.

A noticeable rise in the inner and outer membrane permeability of the tested *S. typhimurium* isolates treated the fenugreek extract was recorded in 40% and 50% of the isolates, respectively (Figure 3).

### 2.3. Antibacterial Action (In Vivo)

The antibacterial impact of the fenugreek extract on the infection caused by *S. typhimurium* was studied in vivo to reveal its clinical significance.

#### 2.3.1. Bacterial Burden and Survival Curve

**Figure 4:**
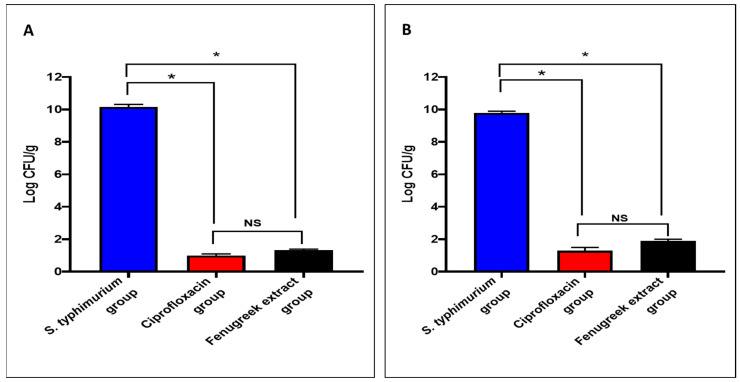
The CFU/mL number in (**A**) the small intestine and (**B**) the caecum. (*) symbolizes a significant difference (*p* < 0.05), and NS denotes a non-significant (*p* > 0.05) difference.

The fenugreek extract produced a noteworthy decrease (*p* < 0.05) in the number of colony-forming units per gram (CFU/g) in the small intestine and caecum tissues (Figure 4).

**Figure 5:**
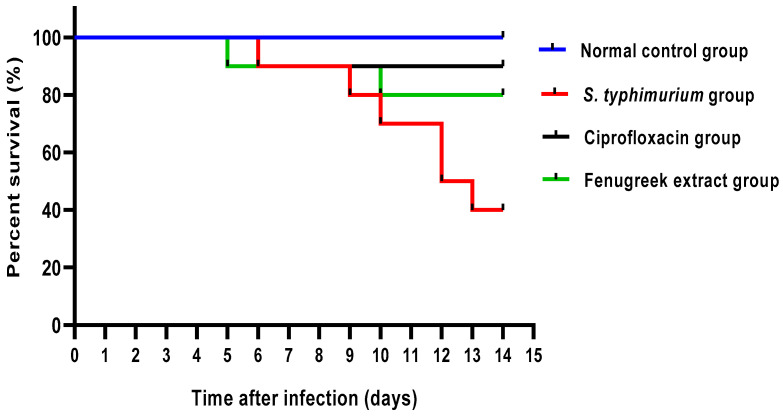
Mice survival curve. All mice were alive till the end of the experiment in the control group. In the *S. typhimurium*-infected group, one mouse died in the sixth, ninth, tenth, and thirteenth days, and two mice died in the twelfth day. In the ciprofloxacin-treated group, only one mouse died on the sixth day. In the fenugreek-extract-treated group, one mouse died on the fifth and tenth days.

As revealed in Figure 5, the fenugreek extract improved the survival rate of the infected mice. All mice in the normal control group were alive till the end of the experiment. In the *S. typhimurium*-infected group, one mouse died on the sixth, ninth, tenth, and thirteenth days, and two died on the twelfth day. Regarding the ciprofloxacin-treated group, one mouse died on the sixth day. In the fenugreek-extract-treated group, only one mouse died on the fifth and tenth days.

#### 2.3.2. Histological Features

**Figure 6:**
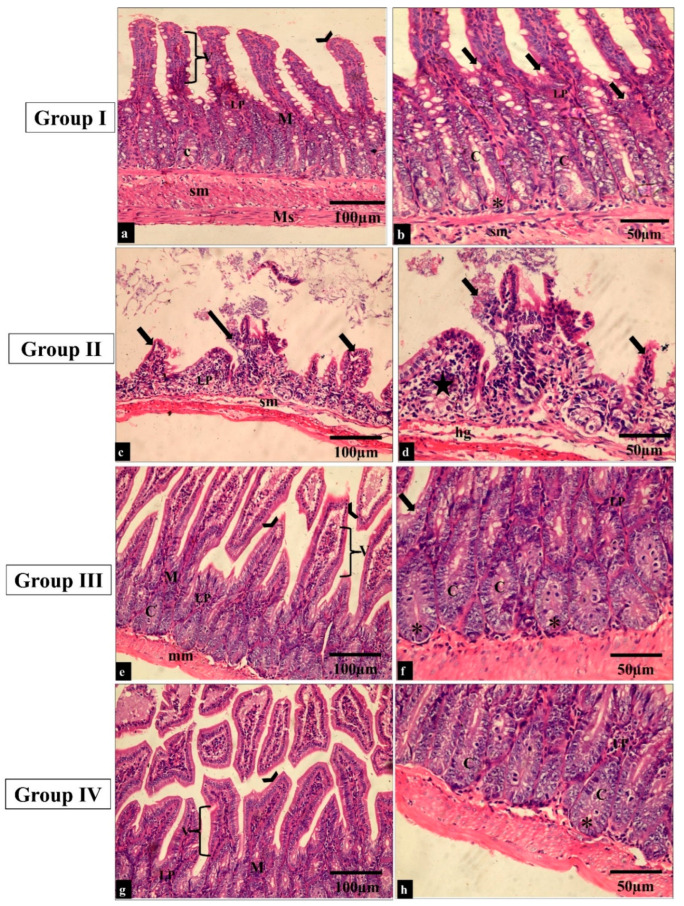
Light microscopic images of H&E-stained sections of the small intestine sections in all the studied groups. (**a**–**f**) Groups I and III show normal histological structure of the small intestine. The small intestine is formed of the mucosa (M), submucosa (sm) and muscularis propria (Ms). The surface epithelial cells are arranged in villi (V) lined by absorptive and goblet cells (thick arrows). The lamina propria (LP) underlies the epithelium; just beneath this is a thin muscularis mucosae of smooth muscle (mm). The intestinal crypts (C) with paneth cells (*) can be seen. (**c**,**b**) Group II shows a shortening of the villi and dense infiltration of the lamina propria by mononuclear cells (star). (**g**,**h**) Group IV shows restoration to the normal histological appearance of the small intestine (H&E × 200, scale bar = 100 μm; H&E × 400, scale bar = 50 μm).

**Figure 7:**
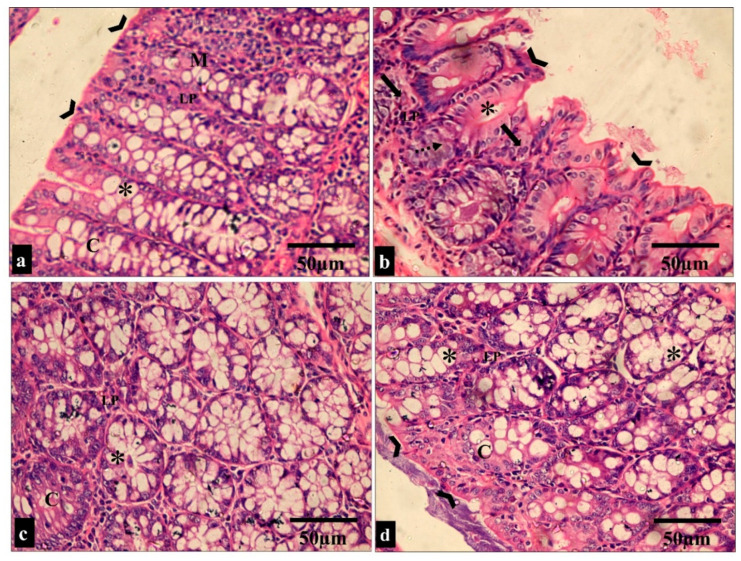
Light microscopic images of the H&E-stained large intestine sections in all the studied groups. (**a**,**c**) Groups I and III show normal colonic mucosa, with the surface of the mucosa covered with a striated brush border (arrowheads). The lamina propria (LP) of the mucosa contains simple tubular intestinal glands (crypts of Lieberkühn) (C). The crypts are lined by numerous goblet cells (*). (**b**) Group II shows loss of surface epithelium (arrowheads) with mononuclear cellular infiltration in the lamina propria of the glandular tissue (arrows). Additionally, there is an apparent reduction in the number of goblet cells (*) and crypt abscess formation (dashed arrow). (**d**) Group IV shows restoration to the normal histological appearance of the colonic mucosa, with straight intact brush borders (arrowheads) and numerous goblet cells in the glandular tissue (*). (H&E × 400, scale bar = 50 μm).

The effect of the treatment with the fenugreek extract on the histological features was investigated after staining the tissues with hematoxylin and eosin (H&E) stain (Figure 6 and Figure 7).

#### 2.3.3. Immunohistochemical Features

**Figure 8:**
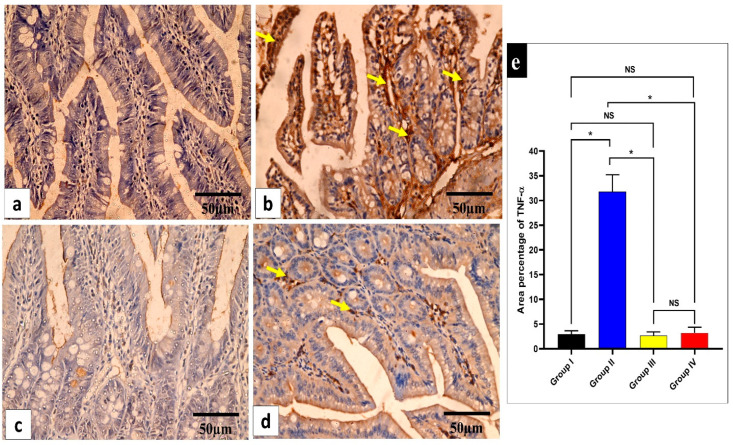
Light microscopic images of TNF-α-stained sections of the small intestine in the different studied groups. (**a**) Group I shows a negative TNF-α immune reaction in the intestinal mucosa cells. (**b**) Group II shows a strong positive TNF-α immune reaction in the intestinal mucosa cells (yellow arrows). (**c**) Group III shows a negative TNF-α immune reaction expression in all intestinal mucosa cells. (**d**) Group IV shows no TNF-α immune reaction in most cells and a weak immune reaction in a few cells (arrows). (**e**) The rea percentage of TNF-α in all the groups. Mean ± standard deviation (SD) is used to express the data. Statistical comparison was performed using a one-way ANOVA and Tukey’s post hoc test for multiple comparisons. The single asterisk represents a significant change, and the abbreviation NS represents a non-significant change (*p* < 0.05). (TNF-α immune reaction in cell immunostaining × 400, scale bar = 50 μm).

**Figure 9:**
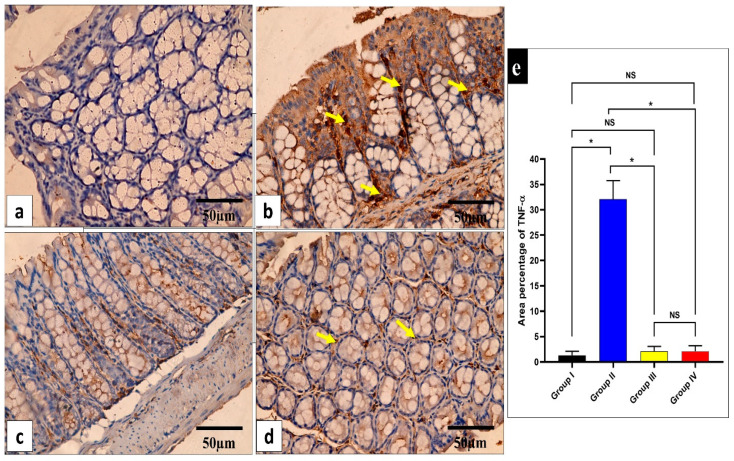
Light microscopic images of TNF-α-stained large intestine sections in the different studied groups. (**a**) Group I shows a negative TNF-α immune reaction in the surface epithelium and all colonic glandular cells. (**b**) Group II shows a strong positive TNF-α immune reaction in the form of a brown cytoplasm of the surface epithelial and glandular cells (yellow arrows). (**c**) Group III exhibits a negative TNF-α immune reaction expression in all mucosal cells. (**d**) Group IV shows no TNF-α immune reaction in the majority of cells and a weak immune reaction in a few glandular cells (arrows). (**e**) The area percentage of TNF-α in all groups. Mean ± SD is used to express the data. Statistical comparison was performed using a one-way ANOVA and Tukey’s post hoc test for multiple comparisons. The single asterisk represents a significant change, and the abbreviation NS represents a non-significant change (*p* < 0.05). (TNF-α immune reaction in cell immunostaining × 400, scale bar = 50 μm).

The influence of the fenugreek extract on the level of the inflammatory marker tumor necrosis factor-alpha (TNF-α) in the studied tissues was investigated (Figure 8 and Figure 9).

#### 2.3.4. ELISA and qRT-PCR

The fenugreek extract was found to significantly (*p* < 0.05) decrease interleukin 6 and 1β (IL-6 and IL-1β), which are proinflammatory interleukins (Table 3).

**Figure 10:**
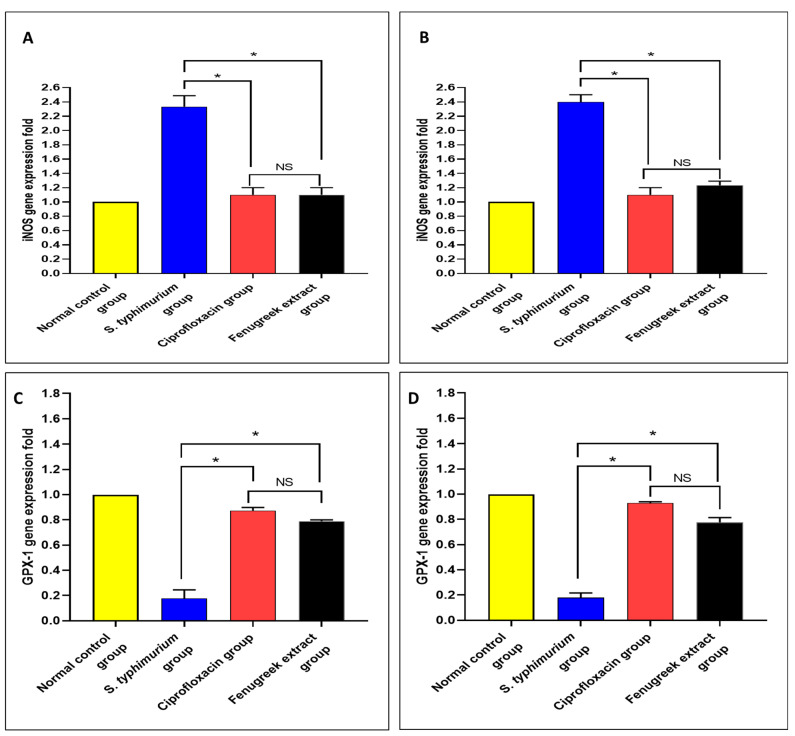
The effect of fenugreek extract on the expression levels of (**A**) iNOS in the small intestine, (**B**) iNOS in the caecum, (**C**) GPX-1 in the small intestine, and (**D**) GPX-1 in the caecum. (*) represents a significant difference (*p* < 0.05), and NS denotes a non-significant (*p* > 0.05) difference.

Using qRT-PCR, the gene expressions of nitric oxide synthase (iNOS) and glutathione peroxidase 1 (GPX-1) were determined (Figure 10). There was a significant reduction (*p* < 0.05) in iNOS gene expression in the fenugreek-treated group. Moreover, there was a significant increase (*p* < 0.05) in the gene expression of GPX-1 in the fenugreek-treated group.

## 3. Discussion

The identification and characterization of the chemical content of *Trigonella foenum-graecum* herb extract was initially performed using LC-ESI-MS/MS, which recorded the existence of 36 compounds of different chemical groups. The initially identified compounds belonged to hydroxy fatty acids, flavonoid glycosides, phenyl propanoic acids, amino acids, purine nucleosides, coumarin, anthocyanidin glycosides, aurone glycoside, and isoflavonoid glycosides. The major compounds were determined as 3,4-dihydroxy benzoic acid, quercetin-3,4’-*O*-di-β-glucopyranoside, caffeic acid, citrulline, 3-hydroxy-3-methyl glutaric acid, acacetin, citraconic acid, isookanin-7-glucoside, esculin, myricetin, luteolin-7-*O*-glucoside, malic acid, and kaempferol-7-neohesperidoside. Several studies have documented the potential antibacterial activity of these bioactive compounds in fenugreek extract [54,55,56,57,58].

Various antibacterials work by disturbing the bacterial membranes to exert their action. The membranes of bacteria are an important barrier that protect them from harmful agents [59,60]. Here, membrane integrity was inspected before and after treatment with the fenugreek extract. A noticeable reduction (*p* < 0.05) in membrane integrity was noticed in 60% of the isolates. The integrity of the bacterial membrane is an indication of its quality. If the membrane is negatively affected, its integrity decreases, and the discharge of DNA and RNA to the bacterial exterior increases [59].

Additionally, membrane permeability is an essential character of the bacterial membrane. Gram-negative bacteria such as *S. typhimurium* have inner and outer membranes [61,62]. We elucidated the impact of fenugreek extract on membrane permeability. Remarkably, the fenugreek extract (*p* < 0.05) significantly augmented inner and outer membrane permeability in 40% and 50% of the *S. typhimurium* bacteria, respectively. These findings suggest that the probable mechanism of action of fenugreek extract on the tested bacteria is related to its detrimental effects on the bacterial membrane.

As previously mentioned, *S. typhimurium* is a common pathogen that can trigger gastroenteritis; therefore, we used a GIT infection model in vivo to illuminate the antibacterial potential of fenugreek extract. Remarkably, there was an improvement in the histological features of the small intestine and caecum after staining with H&E.

The inflammatory mediators in the small intestine and caecum were studied in this study. The immunohistochemical experiments revealed a significant decline (*p* < 0.05) in TNF-α in the fenugreek-treated group. Additionally, using an ELISA, IL-6 and IL-1β were found to considerably decline (*p* < 0.05) in the fenugreek-extract-treated group. TNF-α, IL-6, and IL-1β usually rise in the infected tissues to start the inflammatory process [63,64], which was inhibited by the treatment with fenugreek extract. We utilized qRT-PCR to illuminate the gene expression of iNOS and GPX-1. The fenugreek extract caused a noteworthy reduction in the gene expression of iNOS, which increases the release of reactive oxygen species that trigger cytotoxicity [65]. On the other hand, GPX-1 is an antioxidant enzyme [66], and its gene expression was found to increase significantly in the fenugreek-treated group.

The significant actions of fenugreek may explain its antibacterial effects against *S. typhimurium*. It was found that caffeic acid (a derivative of *p*-hydroxybenzoic) exerted an antimutagenic effect against *Salmonella typhimurium* [67]. Another study showed that caffeic acid, myricetin, and kaempferol have antioxidant, anti-inflammatory, and antiseptic effects [68]. Additionally, *p*-hydroxy benzoic acid and its derivatives negatively affect Gram-positive and Gram-negative bacteria [69]. In addition, *p*-hydroxybenzoic acid showed an anti-inflammatory effect [70,71]. Quercetin, luteolin, and kaempferol glycosides exerted antibacterial activity, as reported by Akroum et al. [72]. Antibacterial effects have also been revealed for citrulline [73], acacetin [74], esculin [75], and anthocyanin as cyanidin glycosides [76].

## 4. Materials and Methods

### 4.1. Collection of Plant Materials and the Formation of the Crude Extract

The herbs (leaves and flowers) of *Trigonella foenum-graecum* were collected from agricultural fields in November 2022 in Tanta, El Gharbia Governorate, Egypt. The collected herbs were desiccated. The dried plants were powdered (2 kg) and macerated in 95% methanol (5 L × 4 times). The combined methanol extract was evaporated using a rotary evaporator under vacuum at 40 °C to give 118.6 gm of the crude residue. Professor El-Sayed Hamid El-Seidy, Faculty of Agriculture, Agronomy Department, Tanta University, authenticated the herb extracts. The Herbarium of the Pharmacognosy Department, Faculty of Pharmacy, Tanta University, own the herb representative sample (PG-A-00123).

### 4.2. LC/ESI-MS/MS of the Fenugreek Herb

#### 4.2.1. Sample Preparation and Injection

The herbs (leaves and flowers) of *T. foenum-graecum* were exposed to LC-ESI-MS/MS analysis, as described by Tsugawa et al. [77]. The sample residue (50 mg) was solubilized in one milliliter of water/methanol/acetonitrile (50:25:25, *v*/*v*%) via vortexing and ultrasonication for two minutes and ten minutes, respectively. Then, 10 µL of a 2.5 µg/mL solution was injected for comparison against a blank sample (the solvent).

#### 4.2.2. Acquisition Method and Analytical Parameters

In-line filter disks (0.5 µm × 3.0 mm, Phenomenex^®^, Torrance, CA, USA) and X select HSS T3 (2.5 µm, 2.1 × 150 mm, Waters^®^, Milford, MA, USA, 40 °C) were employed as a pre-column and analytical column, respectively. The mobile phases were composed of buffer A (5 mM ammonium formate buffer pH 3 with 1% methanol), buffer B (5 mM ammonium formate buffer pH 8 with 1% methanol), and buffer C (100% acetonitrile). The flow rate was set at 0.3 mL/min. The composition of the mobile phase began with 90 (A or B) to 10 (C) for the first 20 min, then inversed from minute 21 to 25, and lastly returned back for the last three minutes until the end at 28 min. Furthermore, the instrument was coupled with the Triple TOF 5600+ (Sciex, Framingham, MA, USA) for IDA acquisition and Analyst TF 1.7.1 (Sciex^®^) for LC-Triple TOF control.

#### 4.2.3. Data Processing

MasterView was utilized for feature (peaks) extraction from the total ion chromatogram (TIC) based on a signal-to-noise ratio of more than five (non-targeted analysis) and sample-to-blank intensities of greater than three. Moreover, a Reifycs Abf (Analysis Base File) Converter (Reifycs^®^, Tokyo, Japan) was employed for Wiff file conversion and MS-DIAL 4.6 (RIKEN^®^ Tokyo, Japan) for data analysis. Metabolite annotation was carried out using the ReSpect Database and fragmentation patterns and retention times mentioned in the previous reports for metabolites isolated from the investigated plant or others.

### 4.3. Bacteria, Chemicals, and Media

Ten *S. typhimurium* bacteria (S1–S10) were included in the current study from the Department of Microbiology and Immunology, Tanta University. The chemicals dimethyl sulfoxide (DMSO), *O*-nitrophenyl-β-galactopyranoside (ONPG), 1-N-phenylnapthylamine (NPN), and methanol were from Merck, Philadelphia, PA, USA, and the media MHA, Muller–Hinton broth (MHB), and MacConkey agar were from Oxoid, Hampshire, UK.

#### 4.3.1. Antibacterial Potential

Using the agar well diffusion method, the antibacterial potential of the fenugreek extract was investigated, as previously reported [77]. Ciprofloxacin was employed as a positive control, and DMSO was a negative control. Fenugreek extract was added to the wells of the Petri dish plates containing MHA, with the tested bacteria spread on their surfaces at a concentration of 2000 µg/mL. The plates were incubated at 37 °C for 24 h and inspected for the appearance of inhibition zones to indicate antibacterial action. Then, the MICs were recorded using the broth microdilution method, as previously explained [78]. In each microtitration plate, the fenugreek extract was serially diluted in MHB from 2048 µg/mL to 0.5 µg/mL, using ciprofloxacin as a positive control and DMSO as a negative control. Bacterial suspensions were added to each well, and the plates were incubated for 18 h at 37 °C. The lowest concentration that hindered bacterial growth was documented as the MIC value.

#### 4.3.2. Impact on the Bacterial Membranes

The impact of fenugreek extract on the membrane integrity of bacteria by measuring the discharge of DNA and RNA, with absorbances at 260 nm, was recorded using a UV spectrophotometer (SHIMADZU, Kyoto, Japan). Additionally, its impact on inner and outer membrane permeability was inspected.

Inner membrane permeability was investigated by tracking the discharge of the β-galactosidase enzyme from the cell by adding ONPG, which is converted by the enzyme to o-nitrophenol (ONP). Then, the absorbance of the formed ONP was measured at 420 nm using an ELISA reader (Sunrise, VA, USA) [79]. The β-galactosidase enzyme under normal conditions is present in the cell interior. When inner membrane integrity is impaired, the enzyme is released from the cell interior to the outside. Upon adding its substrate, ONPG is converted to ONP, which has an absorption at 420 nm.

The outer membrane permeability was investigated using NPN, a fluorescent compound, using a fluorescence spectrophotometer (SHIMADZU, Kyoto, Japan). The fluorescence was detected at 340 and 420 nm [79].

#### 4.3.3. In Vivo Antibacterial Action

The potential antibacterial action of fenugreek extract was revealed in vivo using a GIT infection model using forty male albino mice (22–26 gm). They were provided with filtered water and a standard pellet diet. The experimental protocol was approved by the Research Ethics Committee of the Faculty of Pharmacy, Tanta University, Egypt (number of TP/RE/12/23 p-059).

#### 4.3.4. Experiment Protocol

The animals were divided into four groups (each with ten mice) as follows [80]:

- Normal control (I): not infected.
- *S. typhimurium*-infected group (II): infected with *S. typhimurium* (1 × 10^6^ CFU/mL) via the oral route.
- Ciprofloxacin-treated group (III): infected with *S. typhimurium* and treated with ciprofloxacin (20 mg/kg), taken orally for three constitutive days.
- Fenugreek-extract-treated group (IV): infected with *S. typhimurium* and treated with fenugreek extract (200 mg/kg), taken orally for three constitutive days [81].

The first doses of ciprofloxacin and fenugreek extract were administered after infection within 12 h. Then, mice were monitored for two weeks to calculate their survival rate. After two weeks, the animals were euthanized, and the small intestine and the caecum were withdrawn for histopathological, immunohistochemical, and biochemical studies. Furthermore, the bacterial burden was recorded in the studied tissues by calculating the number of colony-forming units (CFU/g) [62].

#### 4.3.5. Histopathological and Immunohistochemical Investigations

For histological evaluation, the small intestine and caecum of the experimental groups were preserved in 10% formalin saline and prepared for paraffin blocks. Serial sections were obtained on a rotary microtome at 5–6 μm in thickness. Sections were placed in hematoxylin for one minute, washed in water for one minute, and then differentiated in 1% hydrochloric acid ethanol for 10–30 s. Later, they were soaked in water for 20 min, placed in eosin for 5–10 min at room temperature, and then dehydrated using an ethanol gradient. Slides were visualized under a light microscope [82].

Immunohistochemical staining was performed on six-micrometer tissue sections. The sections were dewaxed, rehydrated using a decreasing alcohol series, and then treated with 10% hydrogen peroxide in methanol for 10 min. The sections were then microwaved for 10 min in 0.01 M sodium citrate buffer (pH 6.0), allowed to cool at room temperature, and then given three PBS washes for five minutes. Antigens were recovered by autoclaving in citrate buffer for 11 min following washing. Following this, sections were incubated overnight at 4 °C with primary antibodies. The tissues were then treated for 30 min at room temperature with the rabbit polyclonal TNF-α antibody and 3, 3-diaminobenzidine (Invitrogen, Waltham, MA, USA). Finally, the tissue sections were cleaned in xylene (Sigma, Washington, WA, USA), mounted for visibility, and faintly counterstained with hematoxylin (Sigma, Washington, USA). Slides were observed at ×400 magnification using a light microscope. Morphometric analysis was performed using image analysis tools (Image J, 1.46a, NIH, Bethesda, MD, USA). The mean area percentage of TNF-α protein expression of the different experimental groups was assessed in ten non-overlapping fields of each section at ×400 magnification [83].

#### 4.3.6. Inflammatory and Oxidative Stress Markers Using ELISA and qRT-PCR

IL-6 and IL-1β were quantified in the small intestine and caecum using ELISA kits (Abcam, Cambridge, United Kingdom). The gene expression of iNOS and GPX-1 was determined after extraction of the total RNA using an RNA extraction kit (Invitrogen, Waltham, MA, USA). The cDNA was synthesized, and qRT-PCR was ran using a SYBR green master mix (Qiagen, Hilden, Germany). The employed primers are listed in Table S1.

## 5. Statistical Analysis

Data are shown as mean ± SD after performing the experiments three times. An ANOVA was utilized to evaluate the differences between the groups. If *p* < 0.05, the results were judged to be significant. Kaplan–Meier survival curves were assembled to assess mice survival using the Prism software (GraphPad, La Jolla, CA, USA).

## 6. Conclusions

*Trigonella foenum-graecum* herb extract contains 36 different compounds. The herb exerts antibacterial action on *S. typhimurium* isolates both in vitro and in vivo. This conclusion was attained using various molecular techniques, including ELISA, qRT-PCR, and histopathological and immunohistochemical data. The probable mechanism of action of fenugreek herb extract on the tested bacteria is its harmful impact on the membrane properties of the bacteria. The chemical content of fenugreek herb could explain its antibacterial effect. However, further investigations are crucial to understand the antibacterial mode of action and disclosing its likely influence on other food pathogens, as well as to discovering the underlying mechanism of action.

## Figures and Tables

**Table 1:** Phytochemical analysis of *Trigonella foenum-graecum* using LC-ESI-MS/MS (negative mode).

	RT (min)	Compound Name	Precursor m/z	Error ppm	Formula	MS/MS	Ontology	Reference
1	1.06	3-Hydroxy-3-Methylglutaric acid (meglutol)	161.0457	−0.3	C_6_H_10_O_5_	73.02, 101.02, 116.98, 143.05, 161.04	Hydroxy fatty acids	[23,24]
2	1.09	Malic acid	133.0147	−2.3	C_4_H_6_O_5_	71.013, 88.04, 115.00, 132.88, 133.01	Beta hydroxy acids and derivatives	[25]
3	1.10	Quinic acid	191.0554	1.9	C_7_H_12_O_6_	111.14, 155.01, 164.95, 173.05, 190.95, 191.05	Quinic acid and derivatives	[26]
4	1.11	Myricetin	317.0536	1.5	C_15_H_10_O_8_	151.03, 179.10, 316.88, 317.06	Flavonols	[27]
5	1.14	Caffeic acid	179.059	−12.9	C_9_H_8_O_4_	134.97, 179.05	Hydroxycinnamic acids	[28]
6	1.12	D-3-Phenyllactic acid	165.0401	0.1	C_9_H_10_O_3_	59.01433:107 71.01071:71 72.9928:217 75.00829:600 78.91574:37 89.94, 165.03	Phenyl-propanoic acids	[29]
7	1.19	Citrulline	174.0763	0.2	C_6_H_13_N_3_O_3_	131.07, 173.89, 174.07	L-alpha-amino acids	[30]
8	1.28	Quercitrin	447.114	31.5	C_21_H_20_O_11_	152.01, 300.08, 301.10, 447.128	Flavonoid-3-*O*-glycosides	[31]
9	1.31	Pantothenate	218.1013	1.8	C_9_H_17_NO_5_	71.01, 182.02, 200.09, 218.09	Secondary alcohols	[32]
10	1.62	Citraconic acid	128.958	6.9	C_5_H_6_O_4_	60.99, 68.99, 84.98, 129.00	Methyl-branched fatty acids	[33]
11	1.66	2-Methylglutaric acid	145.0981	−0.7	C_6_H_10_O_4_	87.05, 113.01, 130.06, 145.09	Methyl-branched fatty acids	[34]
12	1.99	Adenosine	266.0899	−5.6	C_10_H_13_N_5_O_4_	107.04, 114.96, 135.04, 202.92, 222.91, 266.08	Purine nucleosides	[35]
13	2.32	Phlorizin	435.1451	−29.5	C_21_H_24_O_10_	57.03, 123.02 167.07 273.07, 435.18	Flavonoid *O*-glycosides	[36]
14	2.58	Isookanin-7-glucoside	449.1079	2.3	C_21_H_22_O_11_	151.00, 287.05, 449.22	Flavonoid-7-*O*-glycosides	[37]
15	3.47	Cyanidin-3-*O*-(2″-*O*-beta-glucopyranosyl-beta-glucopyranoside)	609.1558[M-2H]^−^	−12.1	C_27_H_31_O_16_	218.94, 239.05, 255.02, 285.04, 609.21	Anthocyanidin-3-*O*-glycosides	[38]
16	4.30	Kaempferol-7-neohesperidoside	593.1472	6	C_27_H_30_O_15_	285.02, 477.05, 315.06 593.10, 593.14	Flavonoid-7-*O*-glycosides	[39]
17	4.12	3,4-Dihydroxy benzoic acid	153.0189	0.4	C_7_H_6_O_4_	109.03, 135.00, 153.01	Hydroxybenzoic acid derivatives	[40]
18	4.99	eriodictyol-7-*O*-glucoside	449.105	8.7	C_21_H_22_O_11_	269.09, 287.05, 403.19, 449.19	Flavonoid-7-*O*-glycosides	[41]
19	5.06	Kaempferol-3-*O*-α-L-rhamnoside	431.12	19.7	C_21_H_20_O_10_	89.03, 285.10, 313.07, 395.01, 430.86, 431.11	Flavonoid-3-*O*-glycosides	[42]
20	5.37	Daidzein-8-C-glucoside	415.1895	42.9	C_21_H_20_O_9_	267.13, 295.01, 325.12, 415.198	Isoflavonoid C-glycosides	[43]
21	6.77	Luteolin-7-*O*-glucoside	447.0955	−0.9	C_21_H_20_O_11_	174.95, 151.92, 253.01, 285.03, 447.19	Flavonoid-7-*O*-glycosides	[44]
22	7.16	Maritimetin-6-*O*-glucoside	447.1435	16.3	C_21_H_20_O_11_	133.04, 285.06, 447.27	Aurone O-glycosides	[45]
23	7.69	Kaempferol-3-*O*-robinoside-7-*O*-rhamnoside (robinin)	739.191	−0.1	C_33_H_40_O_19_	283.13, 430.01, 593.12, 739.34	Flavonoid-7-*O*-glycosides	[46]
24	7.97	Cyanidin-3-*O*-galactoside	447.0926[M-2H]^−^	0.1	C_21_H_21_O_11_	150.99, 285.04, 447.25	Anthocyanidin-3-*O*-glycosides	[47]
25	8.41	Daidzein	253.0529	1.2	C_15_H_10_O_4_	135.00, 184.93, 208.93, 225.04, 253.04	Isoflavones	[48]
26	10.69	Datiscin	593.2539	32.4	C_27_H_30_O_15_	285.03, 539.40, 593.25	Flavonoid-3-*O*-glycosides	[39]
27	10.77	Formononetin	267.0353	−16.4	C_16_H_12_O_4_	252.04, 223.91, 267.11	4’-*O*-methylisoflavones	[45]
28	10.92	Apigenin	269.0839	−4.3	C_15_H_10_O_5_	151.00, 225.05, 269.04	Flavones	[45]
29	10.74	Acacetin	283.0617	0.7	C_16_H_12_O_5_	225.05, 252.90, 282.15, 283.05	4’-*O*-methylated flavonoids	[49]
30	11.60	3,5,7-Trihydroxy-4’-methoxyflavone	299.1027	−27.7	C_16_H_12_O_6_	284.03, 299.09	Flavonols	[41]
31	13.27	Luteolin	285.0825	−16.9	C_15_H_10_O_6_	151.03, 256.04, 285.04	Flavones	[44]
32	15.12	Apigenin-7-*O*-glucoside	431.1832	19.7	C_21_H_20_O_10_	268.03, 269.12, 310.91, 431.25	Flavonoid-7-O-glycosides	[45]
33	15.28	Neohesperidin dihydrochalcone	611.1158	6.5	C_28_H_36_O_15_	504.10, 565.11, 611.35	Flavonoid O-glycosides	[50]
34	15.84	Esculin	339.2002	0.8	C_15_H_16_O_9_	179.00, 320.90, 339.19	Coumarin glycosides	[51]
35	15.90	Hesperetin	301.0704	3.9	C_16_H_14_O_6_	174.92, 255.22, 301.06	4’-O-methylated flavonoids	[52]
36	16.77	Quercetin-3,4’-*O*-di-beta-glucopyranoside	625.119	26.5	C_27_H_30_O_17_	301.21, 625.13	Flavonoid-3-O-glycosides	[53]

**Table 2:** MICs of the fenugreek extract on the *S. typhimurium* isolates.

Isolate Code	MIC (µg/mL)
S1	64
S2	256
S3	512
S4	128
S5	128
S6	64
S7	256
S8	128
S9	512
S10	256

**Table 3:** Levels of IL-6 and IL-1β in the studied tissues.

Groups	IL-6 (pg/mg Tissues)		IL-1β (pg/mg Tissues)	
	Small intestine	Caecum	Small intestine	Caecum
Normal control	89.3 ± 3.3 **	93.4 ± 4.6 **	19.7 ± 2.9 **	22.4 ± 1.2 **
*S. typhimurium*-infected group	302.4 ± 7.2	320.3 ± 8.8	98.2 ± 3.7	97.3 ± 4.5
Ciprofloxacin-treated group	95.8 ± 9.2 **	100.4 ± 5.6 **	22.9 ± 4.7 **	25.3 ± 4.6 **
Fenugreek-extract-treated group	100.2 ± 6.7 **	105 ± 6 **	24.5 ± 6.7 **	29.2 ± 3.9 **

(**) represents a significant difference (*p* < 0.05) in relation to the *S. typhimurium*-infected group.

## Data Availability

The data presented in this study are available on request.

## Supplementary Materials

The following supporting information can be downloaded at: https://www.mdpi.com/article/10.3390/ph17020259/s1, Figure S1: Negative mode total ion chromatogram of LC-ESI-MS/MS of Trigonella foenum-graecum herb extract; Table S1: Sequences of the primers.
