# Supplementary material for: Outlining the Phytoconstituents of Greek Clover Herb Extract and Assessment of Its Effect against Foodborne Infections Caused by Salmonella typhimurium
Source: Pharmaceuticals (Basel). 2024 Feb 18;17(2):259. doi: 10.3390/ph17020259 (PMC10892485; doi:10.3390/ph17020259)
Supplement: Supplementary file 1 [file pharmaceuticals-17-00259-s001.zip › pharmaceuticals-2849165-supplementary.pdf]

Phytochemical profiling of Greek clover herb extract and its potential antibacterial effect using *in vitro* and *in vivo* investigations

Jawaher Alqahtani [1,†,*], Walaa A. Negm [2,†], Engy Elekhnawy [3], Ismail A. Hussein [4], Ehssan Moglad [5], Sarah Ibrahim [6], Suzy A. El-Sherbeni [2*]

Figure S1. Negative mode total ion chromatogram of LC-ESI-MS/MS of *Trigonella foenum-graecum* herb extract.

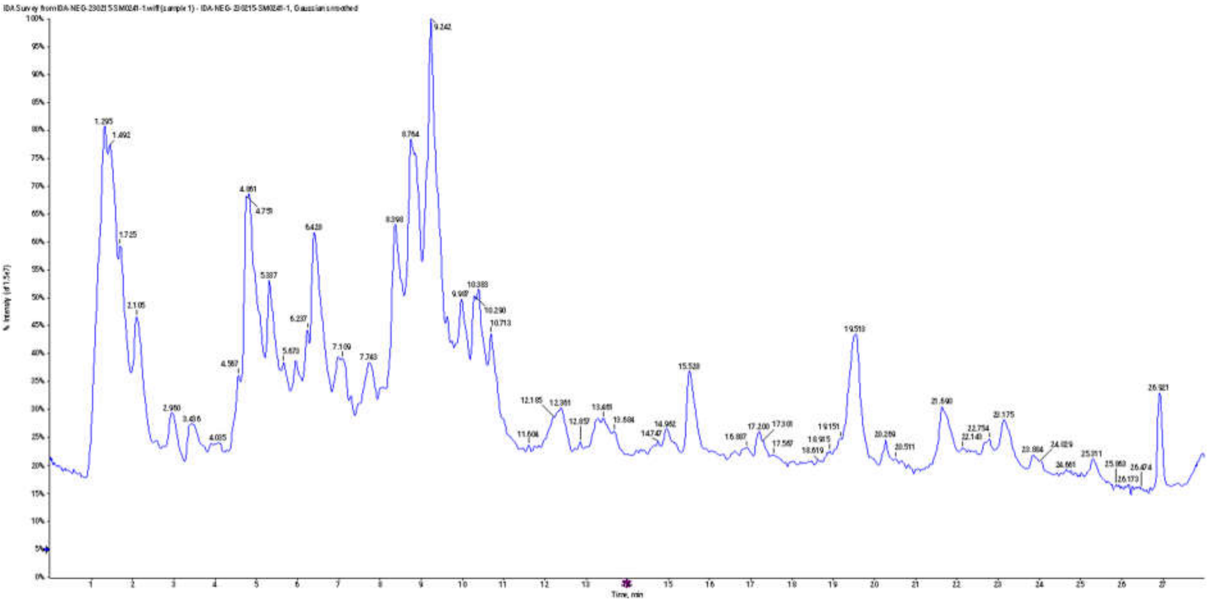

Table S1. Sequences of the primers

Gene	Forward primer	Reverse primer
**iNOS**	GCTATGGCCGCTTTGATGTG	ACCTCCAGTAGCATGTTGGC
**GPX-1**	AATGTCGCGTCTCTCTGAGG	TTGCCATTCTGGTGTCCGAA
**B actin**	GATCAGCAAGCAGGAGTACGA	AAAACGCAGCTCAGTAACAGT
